# Effectiveness of a Web-based Intervention for Problem Drinkers and Reasons for Dropout: Randomized Controlled Trial

**DOI:** 10.2196/jmir.1642

**Published:** 2010-12-16

**Authors:** Marloes G Postel, Hein A de Haan, Elke D ter Huurne, Eni S Becker, Cor AJ de Jong

**Affiliations:** ^3^Radboud University NijmegenBehavioural Science InstituteNijmegenNetherlands; ^2^Tactus Addiction TreatmentEnschedeNetherlands; ^1^Nijmegen Institute for Scientist-Practitioners in AddictionNijmegenNetherlands

**Keywords:** E-therapy, Internet, Online treatment, Alcohol abuse, Substance abuse, web-based, dropout, randomized controlled trial

## Abstract

**Background:**

Online self-help interventions for problem drinkers show promising results, but the effectiveness of online therapy with active involvement of a therapist via the Internet only has not been examined.

**Objective:**

The objective of our study was to evaluate an e-therapy program with active therapeutic involvement for problem drinkers, with the hypotheses that e-therapy would (1) reduce weekly alcohol consumption, and (2) improve health status. Reasons for dropout were also systematically investigated.

**Method:**

In an open randomized controlled trial, Dutch-speaking problem drinkers in the general population were randomly assigned (in blocks of 8, according to a computer-generated random list) to the 3-month e-therapy program (n = 78) or the waiting list control group (n = 78). The e-therapy program consisted of a structured 2-part online treatment program in which the participant and the therapist communicated asynchronously, via the Internet only. Participants in the waiting list control group received “no-reply” email messages once every 2 weeks. The primary outcome measures were (1) the difference in the score on weekly alcohol consumption, and (2) the proportion of participants drinking under the problem drinking limit. Intention-to-treat analyses were performed using multiple imputations to deal with loss to follow-up. A dropout questionnaire was sent to anyone who did not complete the 3-month assessment. Reasons for dropout were independently assessed by the first and third author.

**Results:**

Of the 156 individuals who were randomly assigned, 102 (65%) completed assessment at 3 months. In the intention-to-treat analyses, the e-therapy group (n = 78) showed a significantly greater decrease in alcohol consumption than those in the control group (n = 78) at 3 months. The e-therapy group decreased their mean weekly alcohol consumption by 28.8 units compared with 3.1 units in the control group, a difference in means of 25.6 units on a weekly basis (95% confidence interval 15.69-35.80, *P* < .001). The between-group effect size (pooled SD) was large (d = 1.21). The results also showed that 68% (53/78) of the e-therapy group was drinking less than 15 (females) or 22 (males) units a week, compared with 15% (12/78) in the control group (OR 12.0, number needed to treat 1.9, *P* < .001). Dropout analysis showed that the main reasons for dropouts (n = 54) were personal reasons unrelated to the e-therapy program, discomfort with the treatment protocol, and satisfaction with the positive results achieved.

**Conclusions:**

E-therapy for problem drinking is an effective intervention that can be delivered to a large population who otherwise do not seek help for their drinking problem. Insight into reasons for dropout can help improve e-therapy programs to decrease the number of dropouts. Additional research is needed to directly compare the effectiveness of the e-therapy program with a face-to-face treatment program.

**Trial registration:**

ISRCTN39104853; http://controlled-trials.com/ISRCTN39104853/ISRCTN39104853 (Archived by WebCite at http://www.webcitation.org/5uX1R5xfW)

## Introduction

Problem drinking is a highly prevalent public health issue, with serious consequences in terms of morbidity and mortality [1], and associated economic costs [2] and social problems [3]. However, most problem drinkers will never seek treatment [4]. In the United States, only 16% of people with an alcohol-abuse disorder had received treatment in 2001 [5], and in the Netherlands, only 10% of the problem drinkers received professional help in 2006 [6]. Furthermore, people often seek help only at a late stage; usually after 10 or more years of alcohol abuse or dependence [7]. Therefore, improved access to therapy for problem drinkers is needed [8-10]. The Internet offers a novel opportunity to reach a larger and more diverse segment of the population of problem drinkers [11,12] and improves the availability of alcohol treatment services. Online treatment programs are distinguishable by the intensity of the therapist involvement. Andersson and colleagues [13] distinguished the different forms of Internet interventions in a clear manner: (1) fully self-administered therapy or pure self-help, (2) predominately self-help (ie, therapist assesses and provides initial rationale, and teaches how to use the self-help tool), (3) minimal-contact therapy (ie, active involvement of a therapist, but to a lesser degree than in traditional therapy, eg, using email), and (4) predominantly therapist-administered therapy (ie, regular contact with therapist for a number of sessions, but in conjunction with self-help material). A meta-analysis of 12 randomized controlled trials (RCTs) of Internet-based cognitive behavioral therapy programs for depression and anxiety showed that Internet-based interventions are effective; especially those with therapist involvement [14].

RCTs of Internet interventions for problem drinking are available, and they show promising results [15-23]. However, all of these online alcohol interventions are fully self-help interventions without therapist involvement. The effectiveness of predominantly therapist-administered online therapy for problem drinkers solely via the Internet has not yet been examined in a RCT. It is expected that active therapeutic involvement will lead to greater treatment effects compared with self-help. In addition, we expect to reach another group of people, who prefer intensive personal therapist contact instead of dealing with their problem themselves.

This report describes the main findings from a RCT in which participants were randomly assigned to the 3-month therapist-involved e-therapy program or to the waiting list control group. Because of poor adherence and high dropout rates in e-health interventions [24-26], and a low completion rate (173/527, 33%) in our pilot study [27], we decided to systematically investigate the reasons for dropout as part of our RCT study as well. Insight into those reasons may identify factors that are responsible for dropout, and online treatment programs can consequently be improved to reduce the number of participants ending treatment prematurely. Based on the prior results of our uncontrolled observations, where we found a significant decrease in alcohol consumption and alcohol-related health complaints [27], we tested the hypothesis that e-therapy would (1) reduce weekly alcohol consumption, and (2) improve health status. To our knowledge this is the first RCT that evaluates the effectiveness and reasons for dropout of an e-therapy program for problem drinking with active therapeutic involvement.

## Methods

### Study design and participants

We undertook an open RCT, with recruitment taking place between October and December 2008. To be included in the trial, participants had to be Dutch-speaking problem drinkers in the general population aged 18 years or more. Problem drinking was defined as drinking currently at least 15 units (of 10 g of ethanol) a week for females and 22 units for males, with a maximum of 67 units a week for females and 99 units for males. This was based on the mean weekly alcohol consumption in the pilot study, added with 1.5 SD. We excluded participants treated for problem drinking in the preceding year and participants with psychiatric treatment in the past 6 months or those currently having a psychiatric disorder.

Participants were recruited through an advertisement on the website’s homepage (http://www.alcoholdebaas.nl), through media attention on national television, and by responding to 500 expressions of interest that had been emailed to the website. Participants were referred to a research website for additional information about the study and encouraged to screen themselves on the inclusion criteria. A total of 169 participants deemed themselves eligible, provided online informed consent, and completed the baseline questionnaire. Participants received the e-therapy intervention free of charge. We did not provide any kind of incentive for study participation. The study protocol was approved by the independent medical ethics board METiGG (ref. no. NL20742.097.07) and registered at http://www.controlled-trials.com (ISRCTN39104853).

### Procedure

**Figure 1:**
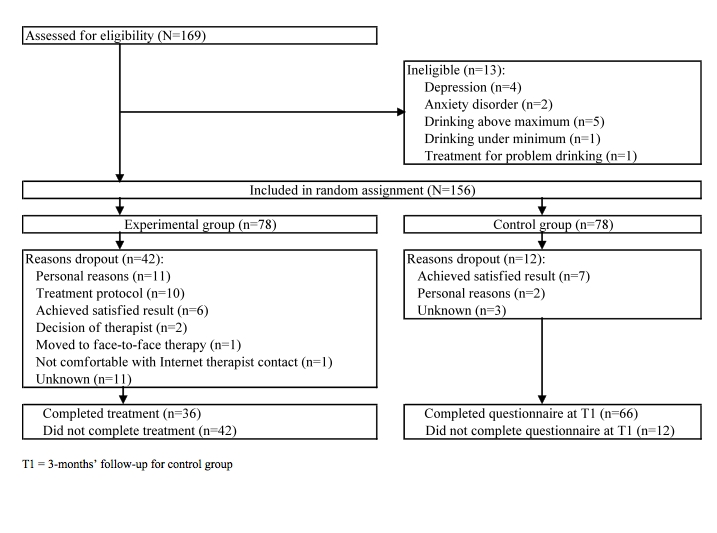
CONSORT diagram: flow of participants through the study protocol.

As shown in the flow chart (Figure 1), 156 of the 169 participants screened were subsequently determined to be eligible for the study and were randomly assigned to either the e-therapy treatment group or to the waiting list control group. Participants were randomly assigned in blocks of 8, according to a computer-generated random list (based on a random generator and algorithm, Microsoft .NET Framework version 3, Microsoft, Bellevue, WA, USA), implemented by a technician who was not involved in the recruitment process. Block randomization ensures group numbers are evenly balanced at the end of each block. Because of the limited availability of the therapists, we needed to keep the numbers in both groups very close at all times. Participants were automatically allocated by computer.

Every e-therapy participant was assigned to a personal therapist for the duration of the study. The 12 experienced therapists were all qualified social workers with higher vocational education, who had received special training in the technical aspects and content of the e-therapy program, with special focus on motivational writing skills. Therapists could obtain expert advice from the multidisciplinary team, consisting of treatment staff, an addiction medicine specialist, a psychologist, and 2 supervisors. Both supervisors regularly checked the therapists’ files for fidelity to treatment protocols. Participants were allocated on a sequential basis to the next available therapist. The mean total time spent on each participant was approximately 1.5 hours per week.

### Interventions

**Figure 2:**
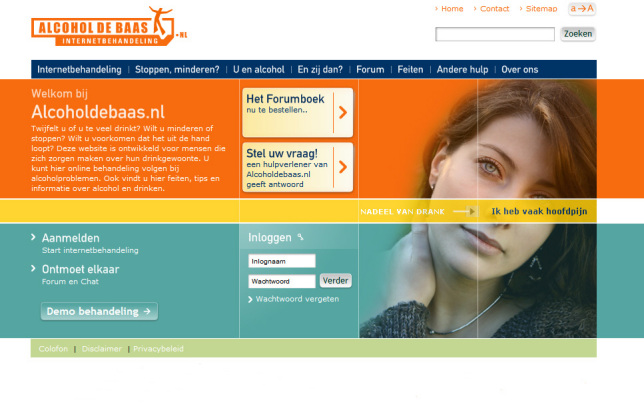
Homepage of http://www.alcoholdebaas.nl

**Figure 3:**
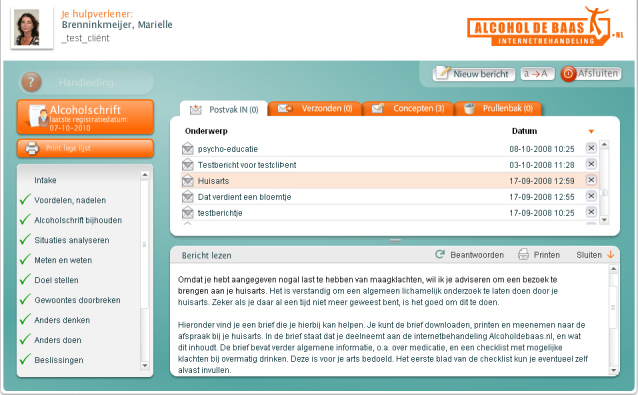
Participant’s personal record

The e-therapy program could be accessed via the homepage (Figure 2) and consisted of a structured 2-part online treatment program in which the participant and the therapist communicated asynchronously, via the Internet only. Participants accessed the e-therapy program in their personal environment. Participant and therapist were in separate or remote locations; the interaction occurred with a time delay between the responses. The aim of the e-therapy program was to reduce or stop the participant’s alcohol intake. The method underlying the program was based on the principles of cognitive behavior therapy [28] and motivational interviewing [29]. All communication between therapists and participants took place through a Web-based application (Figure 3), as described previously [27].

Part 1 of the program consisted of 2 assessments and 4 assignments, with the accompanying communication focusing on the analysis of the participants’ drinking habits. Part 1 covered the following core concepts: (1) exploring advantages and disadvantages of alcohol use, (2+3) understanding drinking patterns through completion of a daily drinking diary and descriptions of the craving moments, and (4) identifying risky drinking situations. The therapist helped the participant at every step in the program; he or she explained the assignments and provided feedback. The therapist always responded within 3 days. Messages were always personalized, although therapists used preprogrammed text parts for the analogous parts, such as the explanation of an assignment. The therapist and participant could not move on to the next assignment until they completed the previous one. We chose a linear model, also called tunnel IA design, as the therapy program is most effective with a specific ordering of treatment steps, and this model is also useful in working with homework assignments and tailored feedback [30]. The therapist provided contact details of the institution that participants could reach 24 hours a day in case of crisis situations. At the end of part 1, personalized advice was given and the participant could choose whether to continue with treatment in part 2 or to stop. The multidisciplinary team evaluated every participant’s record and gave advice to the therapist for the further treatment stages in part 2.

Part 2 focused on behavioral change and included 5 central concepts: (1) setting a drinking goal, which could be abstinence or moderate drinking, (2) formulating helpful and nonhelpful thoughts, (3) considering helpful behaviors for moments of craving, (4) identifying the moment of the decision to drink alcohol, and (5) formulating an action plan for maintaining the new drinking behavior and for preventing relapse. The mean duration of the total e-therapy program was 3 months, with 1 or 2 therapist contacts per week and daily self-registration during the whole program. Besides registration, the participant usually responded every 3 or 4 days. If there was no response from the participant, the therapist contacted the participant 3 times during the following 2 weeks. If there was still no response, the participant received a message that his or her record would be closed after 2 weeks. The posttreatment questionnaire was sent to the participant’s personal data record.

Participants in the waiting list control group received “no-reply” email messages once every 2 weeks during the waiting period of 3 months to keep them involved in the study. The messages contained alcohol-related information, psychoeducational material, motivational messages, and references to the information website and the forum for online contact with fellow sufferers. Participants knew that they were assigned to the control group and that they could start the e-therapy intervention after they completed the assessment at 3 months.

### Outcome measures

All data were collected online. Participants completed online self-report questionnaires at baseline and at 3-months’ follow-up (control group) or at posttreatment, which was at approximately 3 months (e-therapy group). Weekly alcohol consumption was assessed by a 7-day retrospective drinking diary [31]. Type and severity of substance dependence was assessed by the Substance Abuse Module of the Composite International Diagnostic Interview [32]. The General Health Questionnaire (GHQ-28) and the Maudsley Addiction Profile, Health Symptom Scale (MAP-HSS) were used to assess health status [33,34]. The 21-item Depression Anxiety Stress Scale (DASS-21) was used to measure the 3 related negative emotional states of depression, anxiety, and stress [35]. Quality of life was measured with the EuroQol-5D (EQ-5D) [36] and initial treatment motivation with the TCU Motivation for Treatment (MfT) scale [37]. To determine the reasons for dropout, we sent an email to all dropouts with a link to an additional online questionnaire consisting mainly of open questions. If participants did not complete this questionnaire, they were contacted by telephone to remind them to complete the questionnaire online or to administer it by phone immediately. Dropout was defined as anyone who did not complete the 3-month assessment. Dropouts in the e-therapy group did not complete all 12 treatment sessions: 9 assignments and 3 assessments. Because of the design of the e-therapy program it was impossible for participants to skip parts of the intervention; therefore, adherence corresponds to the moment of dropout.

The primary outcome measures were (1) the difference in the score on weekly alcohol consumption, and (2) the proportion of participants drinking under the problem drinking limit. Secondary outcomes were difference scores on health status (GHQ-28 and MAP-HSS), DASS-21 scores, and quality-of-life ratings (EQ-5D).

### Sample size and statistical analysis

Based on the results of our explorative study, we anticipated a 50% reduction of mean weekly alcohol consumption in the experimental group and 25% in the control group. To detect a difference of 25% with an alpha of .05 and a power of 80%, 45 participants were required in each group. To allow for dropouts, our target sample size was 75 participants in each group.

We used chi-square and *t* tests for demographic data and pretreatment characteristics to assess whether randomization resulted in 2 comparable groups at baseline and whether any differential loss to follow-up had occurred. We performed intention-to-treat analysis using multiple imputations (SPSS version 17.0, SPSS Inc, Chicago, IL, USA) to deal with loss to follow-up. We used 5 imputed data sets, and group was used as predictor in the imputation equation. We used *t* tests to assess the differences between pre- and posttreatment measures. Between-group effect sizes were calculated based on the pooled standard deviation, Cohen d. Effect sizes of .80 were considered to be large [38].

Reasons for dropout were independently assessed by the first and third author. If the 2 authors did not agree, the topic was discussed to reach agreement. If necessary, the second author was consulted to arbitrate.

## Results

### Participant characteristics

Table 1 presents baseline characteristics of the 156 participants who were included in the trial. Of these, 54% were women, 58% had a higher education level, and 82% were employed; age ranged from 22 to 66 years, with a mean of 45.3 years. A total of 127 participants reported alcohol dependence (81%). The majority (134/156, 86%) had never received professional help for their drinking problem. The mean weekly alcohol consumption was 41.9 standard units a week: 49.8 for men and 35.2 for women. Participants used a considerable amount of medication for somatic complaints, but no medication that interfered with the treatment program, with the exception of one person using anticraving medication.

Chi-square analysis indicated that there was a significant difference between the groups on prior alcohol treatment; the experimental group had received more alcohol addiction treatment than the control group (24% vs 4%, X^2^_1_ = 13.5, *P* < .001). There were no other significant differences in treatment condition in any of the variables presented in Table 1.

**Table 1:** Baseline characteristics of test populations

Variable		E-therapy Group		Control Group		Total		Analysis		
		(n = 78)		(n = 78)		(N = 156)				
		n	%	n	%	n	%	X^2^	df	*P*
Female		42	54	42	54	84	53.8	0.0	1	1.00
Higher education		42	54	48	62	90	57.7	0.9	1	.33
Employed		65	83	63	81	128	82.1	0.2	1	.68
**DSM-IV ^a^ diagnoses**								1.1	2	.56
	Alcohol dependence	65	83	62	79	127	81.4			
	Alcohol abuse	6	8	10	13	16	10.3			
	No dependence or abuse	7	9	6	8	13	8.3			
Prior alcohol treatment		19	24	3	4	22	14.1	13.5	1	<.001
Problem drinking ^b^		78	100	78	100	156	100	0.00	1	1.00
									
		Mean	SD	Mean	SD	Mean	SD	*t*	df	*P*
Age (years)		46.7	9.7	43.9	9.7	45.3	9.8	1.8	1,154	.08
**Weekly alcohol consumption**										
	Males	47.6	21.3	51.9	16.7	49.8	19.1	-1.0	1,70	.34
	Females	36.3	13.0	34.1	14.5	35.2	13.7	0.7	1,82	.46
GHQ-28 score ^c^		53.6	12.1	55.6	11.7	54.6	11.9	-1.1	1,154	.28
MAP-HSS score (0-40) ^d^		20.3	6.6	20.0	5.3	20.2	6.0	0.3	1,148	.76
DASS-21 total score ^e^		27.5	20.0	28.4	14.7	27.9	17.5	-0.3	1,154	.75
**MfT subscales ^f^**										
	Recognition of General Problems	3.6	0.8	3.5	0.6	3.5	0.7	0.6	1,145	.58
	Recognition of Specific Problems	2.2	0.7	2.2	0.5	2.2	0.6	-0.2	1,143	.86
	Desire for Help	3.9	0.7	3.9	0.6	3.9	0.7	0.5	1,154	.63
	Treatment Readiness	4.1	0.5	4.0	0.4	4.1	0.5	0.8	1,154	.45
EQ VAS ^g^		60.2	22.3	59.7	21.8	59.9	22.0	0.1	1,154	.90

^a^ Diagnostic and Statistical Manual of Mental Disorders, 4th revision

^b^ Drinking >21 (male) or >14 (female) units mean per week

^c^ General Health Questionnaire

^d^ Maudsley Addiction Profile, Health Symptom Scale

^e^ Depression Anxiety Stress Scale

^f^ TCU Motivation for Treatment scale

^g^ EuroQol-5D visual analog scale

### Loss to follow-up

Of the 156 individuals who were randomly assigned, 102 (65%) completed assessment at 3 months (Figure 1). Loss to follow-up at 3 months was higher in the e-therapy group (42/78, 54%) than in the control group (12/78, 15%, X^2^_1_ = 25.5, *P* < .001). Completers and noncompleters in the e-therapy condition differed in 1 variable at baseline: the mean score on the Treatment Readiness subscale of the MfT was higher for completers (mean = 4.23) than for noncompleters (mean = 3.98, F_1,76_ = 5.89, *P* = .02). In the control condition the groups differed in 2 variables: more noncompleters were male (92% vs 38%, X^2^_1_ = 11.82, *P* < .001) and fewer of them had a diagnosis of alcohol dependence (58% vs 83%, X^2^_1_ = 3.89, *P* = .04).

### Outcome

Participants allocated to the e-therapy group showed a greater decrease in alcohol consumption than those in the control group at 3 months (Table 2). The e-therapy group significantly decreased their mean weekly alcohol consumption by 28.8 units compared with 3.1 units in the control group, a difference in means of 25.6 units on a weekly basis (95% confidence interval [CI] 15.69-35.80; *P* < .001). The between-group effect size (pooled SD) was large (d = 1.21). Additional analyses showed no effect modification and confounding for gender and prior alcohol treatment (data not shown).

**Table 2:** Difference scores by treatment condition at 3 months

	E-therapy (n = 78)		Control (n = 78)		Analysis			
Measure	Mean	SD	Mean	SD	95% CI		*P*	Effect size
Weekly alcohol consumption	28.8	21.3	3.1	21.2	25.65 (15.69-35.80)		<.001	1.21
MAP-HSS score (0-40) ^a^	5.2	5.2	0.9	3.7	4.27 (2.37-6.17)		<.001	0.96
GHQ-28 score ^b^	12.8	12.0	4.3	10.4	8.46 (3.82-13.09)		<.005	0.76
DASS-21 total score ^c^	16.3	19.4	2.2	15.6	14.13 (7.96-20.29)		<.001	0.81
EQ VAS ^d^	-10.6	29.4	-2.7	25.6	-7.95 (-16.69 to 0.79)		0.08	-0.29
								
	n	% success	n	% success	OR	NNT ^e^	*P*	
Drinking within guidelines	78	68%	78	15%	12.04	1.9	<.001	

^a^ Maudsley Addiction Profile, Health Symptom Scale

^b^ General Health Questionnaire

^c^ Depression Anxiety Stress Scale

^d^ EuroQol-5D visual analog scale

^e^ Number needed to treat

The clinical significance of the e-therapy program was assessed using the number of participants with alcohol consumption under the problem drinking limit at 3 months. The results showed that 68% of the e-therapy group was drinking less than 15 (females) or 22 (males) units a week, compared with 15% in the control group (OR 12.0, number needed to treat 1.9, *P* < .001).

The secondary outcome data showed that participants in the e-therapy group scored significantly better on the MAP-HSS (95% CI 2.37-6.17, *P* < .001), GHQ-28 (95% CI 3.82-13.09, *P* < .005), and DASS-21 (95% CI 7.96-20.29, *P* < .001), but not on the EQ-5D (Table 2).

### Compliance

**Figure 4:**
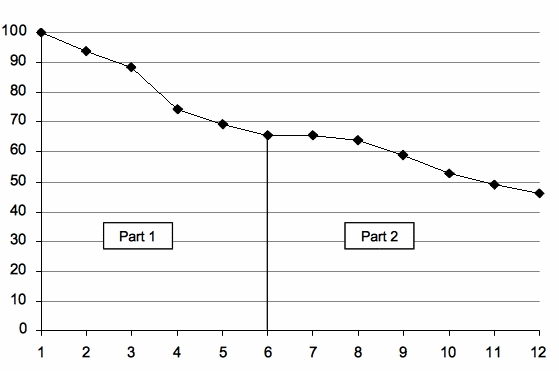
Attrition curve e-therapy group: proportion participants by number of assignments

In the e-therapy group, the mean number of sessions completed was 8.3 (SD 4.2) out of 12. Participants completed the modules in the order that they were presented. Treatment completers (36/78, 46%) completed all 12 assignments and dropouts (n = 42) completed a mean of 5.1 (SD 3.2) assignments. The dropout rate was higher in part 1 (36%) than in part 2 (19%). Figure 4 shows the attrition curve for the e-therapy group. The mean duration of treatment completion was 16.6 weeks and the mean waiting time of the control group was 14.2 weeks.

### Reasons for dropout

A substantial number of participants in the e-therapy group (n = 42) and in the control group (n = 12) did not complete postassessment. We were not able to contact 14 participants, because of nonresponse or an invalid phone number. However, we could establish that in the e-therapy group 11 participants dropped out because of personal reasons unrelated to the e-therapy program or the study (eg, ill family member), 10 because they were not comfortable with the treatment protocol (eg, too intensive), and 6 because they were satisfied with the positive results being achieved (eg, “I have been sufficiently helped”). Additionally, 1 person was not comfortable with the Internet therapist contact, 1 participant moved on to face-to-face treatment, and the therapist decided to terminate the e-therapy on 2 occasions, 1 because of insufficient information and the other due to an inability to set a realistic drinking goal. In the control group, 7 participants quit because they were satisfied with the results achieved and 2 for personal reasons.

## Discussion

### Main results

Participants who received the therapist-supported e-therapy program reported substantially greater gains than those who received no-reply email messages. At the end of treatment, 7 out of 10 participants in the e-therapy group achieved drinking behavior within the guidelines for low-risk drinking. The e-therapy group also showed greater improvement than the control group on general health and depression symptoms. Besides the outcome measures, this study also gained insight into the reasons for dropout; the main reasons for dropping out of the e-therapy program were personal reasons unrelated to the program, the protocol or content of the e-therapy program, and satisfaction with the positive results that had been achieved.

### E-therapy with active therapeutic involvement

This is, to our knowledge, the first RCT evaluating an online treatment program with active therapeutic involvement for problem drinking solely via the Internet. The results of the present study replicate the results of our uncontrolled observations [27]. The effect sizes in this study are quite large compared with effects found for other Web-based interventions designed to decrease alcohol consumption [15,25]. A possible explanation might be the active therapeutic involvement in the present intervention, which replicates earlier findings from Spek et al [14] that active therapeutic involvement seems to be especially effective. It also seems reasonable that the large effects are a result of the key ingredients of the e-therapy program: the therapy itself was intensive; the therapists were experienced, were well educated, and had special training and good supervision throughout the trial; and the recruitment process involves a certain amount of motivation and readiness to change. Further research is needed to identify the effective elements of the e-therapy program and the optimal amount of therapeutic contact needed.

Although around 80% of participants were deemed to be dependent drinkers by Diagnostic and Statistical Manual of Mental Disorders, 4th revision (DSM-IV), it may be that the severity of dependence was actually quite low, as a high proportion of the participants were employed and well educated.

E-therapy attracts participants who are otherwise unlikely to use regular face-to-face treatment facilities or self-help programs. A study by Postel et al [12] showed that e-therapy reaches more women, higher-educated people, and employed people, groups that are underrepresented in regular face-to-face therapy. One of the perceived advantages of e-therapy over a face-to-face treatment is its anonymity. Participants no longer need to stay away from treatment because of shame, fear of stigmatization, or another high barrier to professional help. Furthermore, e-therapy helps participants in their own environment at a time of their own choosing; they no longer need to visit the therapists’ office for scheduled weekly visits, which makes e-therapy more easily accessible and convenient. This is also the reason for choosing asynchronous communication instead of chat; using chat these advantages would no longer exist. An advantage of active therapeutic involvement over self-help is the added value of personal contact with the professional therapist. Although (tailored) screening or self-help interventions have proven to be successful [10,16-18,39], some participants prefer having contact with a professional therapist. Based on the findings of online treatment for depression and anxiety [14], online treatment with therapist involvement might also be more effective than online self-help for alcohol problems.

### Dropout

The dropout rate in this study was substantial (54/156, 35%). E-therapy dropouts showed less readiness for treatment. It is important to note that there were more dropouts in the e-therapy group (42/78, 54%) than in the control group (12/78, 15%), which suggests that actively working on behavioral change causes more resistance and fear than waiting for change. This corresponds to our experiences in regular addiction health care practice, where we see that as patients embark on changing their addictive behavior, it is the fear that dominates. On the other hand, the intention to change your alcohol consumption in the near future is ego syntonic. This might explain the differences in dropout rate between the 2 groups, and this may also be the reason for the overall high dropout rate in addiction treatment interventions.

Although e-therapy is suitable for a broad range of participants, it probably will not be the best alternative for each problem drinker. Some problem drinkers prefer real-life contact with their therapist, and for some participants another form of treatment is recommended because of their specific situation.

The main reasons for dropout in our study are in line with earlier findings on potential factors for attrition as described in the law of attrition by Eysenbach [26]. Personal reasons unrelated to the e-therapy program fall under “external events,” and not being comfortable with the treatment protocol falls under “workload and time required.” However, satisfaction with the positive results being achieved seems to be a new factor, not yet covered in the law of attrition. Eysenbach describes “tangible and intangible observable advantages in completing the trial or continuing to use it” as a potential factor, which refers to advantages when completing the trial or intervention. In our study, participants mentioned a different thing: since they already achieved their treatment goal during the intervention, they decided that completing the trial or continuing to use the intervention would not lead to additional advantages. It seems that some of the e-therapy participants who did not complete the entire program received what they considered to be enough therapy. It would be interesting to confirm this hypothesis, although we realize that it is difficult to obtain data from dropouts. Instead of sending a separate dropout questionnaire, the participants’ situation could be monitored more closely by using interim questionnaires to measure more frequently during the study. Another possibility is to develop the daily registration tool (eg, drinking diary) in a way that data can easily be transported for research purposes.

### Methodological considerations

Despite randomization, a substantially higher proportion of participants in the e-therapy group than in the control group received prior alcohol treatment. Therefore, part of the reduction in alcohol consumption might be explained by this baseline difference. Prior alcohol treatment has been shown to have predictive power with regard to treatment outcome; however, other studies have shown the reverse [40]. Although the large differences between both groups already suggested that prior treatment would play no meaningful role in our study, we performed additional analyses and revealed that prior alcohol treatment had no significant effect on treatment outcome.

Although high dropout rates seem to be characteristic of online interventions [24], this highlights a weakness in our study; especially as we were not able to acquire posttest data from the dropouts as a consequence of the technical procedures of the e-therapy program. We therefore could contact dropouts only by a dropout questionnaire sent separately by email. In future studies, procedures will be changed to ensure that posttreatment assessment can be completed, independent of treatment completion.

We consider the formal investigation of the reasons for dropout to be a strength of our study, as only 1 previous study has formally examined the reasons for dropout [24,41]. This study from Lange and colleagues studied online therapy for posttraumatic stress disorder and showed that the 2 reasons for quitting were technical problems and the form and content of the therapy [41]. As their study was conducted in 2003, and computer and Internet technology has significantly improved since then, it could be expected that technical aspects would no longer one of the main problems. In line with Lange and colleagues, we also found that dissatisfaction with the form or content of the e-therapy program is a reason for dropout. In addition to their findings, we also found that personal reasons and satisfaction with the results achieved were reasons for dropout. Contrary to our expectations, our results show that quitting the e-therapy program prematurely does not automatically mean that the participant has relapsed. Satisfaction with the results being achieved for 7 participants in the control group suggests that receiving informational email messages can be very helpful for some participants. This is most likely true for the group with less serious alcohol problems, as fewer dropouts in the control group had a diagnosis of alcohol dependence. Based on the information on dropout, the e-therapy program can be improved to decrease the number of participants dropping out.

We expect to be able to generalize the 3-month findings of our study to the general population of e-therapy clients, as our sample was comprehensively representative. We kept the exclusion criteria to a minimum, and therefore reached a population of problem drinkers that shows many similarities with participants in the daily practice open-access intervention of the e-therapy program.

We can only report short-term effects of the e-therapy intervention. It was not possible to compare group outcomes at 6 months because of a prior decision to permit the waiting list controls to receive e-therapy after 3 months; this was done for ethical reasons. We know that this is a serious study limitation, as it is important to know the longer-term effects of alcohol treatment programs. A study from Riper and colleagues [42] showed that the beneficial effect of their online alcohol self-help intervention had disappeared at 12 months.

### Future directions and implications

Until recently, the e-therapy program had been available only in Dutch. However since February 2010, the e-therapy program is also available in English (http://www.lookatyourdrinking.com). This greatly expands the implementation of this e-therapy program, and offers the possibility to reach a larger population of problem drinkers and to conduct cross-cultural research. Although the Dutch version of the e-therapy program is fully reimbursed by the health insurance companies and therefore free of charge for participants, the English version unfortunately is not yet. English participants have to pay for the treatment program themselves.

Insight into the reasons for dropout offers possibilities for the improvement of online treatment programs. For example, more therapist attention for participants’ satisfaction will possibly result in more treatment terminations in good consultation. Sending an email alert to participants when they receive a new message from their therapist can easily eliminate part of the dissatisfaction. At this moment, the challenge of e-therapy programs no longer seems to be its effectiveness but keeping participants involved till the end of the treatment program.

In summary, it appears that, because many problem drinkers do not receive any kind of treatment, these initial results point to a meaningful way to deliver easily accessible and effective alcohol treatment to a larger population, members of which do not otherwise seek or receive help for their drinking problem. Additional research is needed to gain more insight into reasons for dropout and to directly compare the effectiveness of the e-therapy program with a face-to-face treatment program. We plan to conduct secondary analysis after treatment completion in both groups. We will then merge the experimental and control groups to explore whether e-therapy might work more effectively for some people than for others.

## Abbreviations

CI: confidence interval
DASS-21: Depression Anxiety Stress Scale
DSM-IV: Diagnostic and Statistical Manual of Mental Disorders, 4th revision
EQ-5D: EuroQol-5D
GHQ-28: General Health Questionnaire
MAP-HSS: Maudsley Addiction Profile, Health Symptom Scale
MfT: TCU Motivation for Treatment scale
RCT: randomized controlled trial
